# State and national household concentrations of PM_2.5_ from solid cookfuel use: Results from measurements and modeling in India for estimation of the global burden of disease

**DOI:** 10.1186/1476-069X-12-77

**Published:** 2013-09-11

**Authors:** Kalpana Balakrishnan, Santu Ghosh, Bhaswati Ganguli, Sankar Sambandam, Nigel Bruce, Douglas F Barnes, Kirk R Smith

**Affiliations:** 1 Department of Environmental Health Engineering, Sri Ramachandra University, Chennai, India; 2 Department of Statistics, University of Calcutta, Kolkata, India; 3 University of Liverpool, Liverpool, UK; 4 Energy for Development, Washington D.C., USA; 5 Division of Environmental Health Sciences, School of Public Health ,University Of California, Berkeley, USA

**Keywords:** Household air pollution, Exposure assessment, Comparative risk assessment, National Family Health Survey(NFHS), Log-linear regression models

## Abstract

**Background:**

Previous global burden of disease (GBD) estimates for household air pollution (HAP) from solid cookfuel use were based on categorical indicators of exposure. Recent progress in GBD methodologies that use integrated–exposure–response (IER) curves for combustion particles required the development of models to quantitatively estimate average HAP levels experienced by large populations. Such models can also serve to inform public health intervention efforts. Thus, we developed a model to estimate national household concentrations of PM_2.5_ from solid cookfuel use in India, together with estimates for 29 states.

**Methods:**

We monitored 24-hr household concentrations of PM_2.5_, in 617 rural households from 4 states in India on a cross-sectional basis between November 2004 and March 2005. We then, developed log-linear regression models that predict household concentrations as a function of multiple, independent household level variables available in national household surveys and generated national / state estimates using The Indian National Family and Health Survey (NFHS 2005).

**Results:**

The measured mean 24-hr concentration of PM_2.5_ in solid cookfuel using households ranged from 163 μg/m^3^ (95% CI: 143,183; median 106; IQR: 191) in the living area to 609 μg/m^3^ (95% CI: 547,671; median: 472; IQR: 734) in the kitchen area. Fuel type, kitchen type, ventilation, geographical location and cooking duration were found to be significant predictors of PM_2.5_ concentrations in the household model. k-fold cross validation showed a fair degree of correlation (r = 0.56) between modeled and measured values. Extrapolation of the household results by state to all solid cookfuel-using households in India, covered by NFHS 2005, resulted in a modeled estimate of 450 μg/m^3^ (95% CI: 318,640) and 113 μg/m^3^ (95% CI: 102,127) , for national average 24-hr PM_2.5_ concentrations in the kitchen and living areas respectively.

**Conclusions:**

The model affords substantial improvement over commonly used exposure indicators such as “percent solid cookfuel use” in HAP disease burden assessments, by providing some of the first estimates of national average HAP levels experienced in India. Model estimates also add considerable strength of evidence for framing and implementation of intervention efforts at the state and national levels.

## Background

The high prevalence of solid cookfuel use (such as biomass and coal) for household energy needs in poor communities of developing countries [1,2] has been known to result in exposures to multiple toxic products of incomplete combustion and is amongst the leading environmental risk factors contributing to the global burden of disease [3,4]. The over 200 studies that have measured air pollution levels in developing country households, across all WHO regions [5] provide unequivocal evidence of extreme exposures in solid cookfuel using settings, often many fold higher than recommended WHO Air Quality Guidelines (AQGs)[6].

Household concentrations and personal exposures to air pollutants resulting from solid cookfuel combustion can vary according to a hierarchy of factors. Several studies [7-13] have shown the distribution of exposures to be heterogeneous and complex with multiple determinants (such as fuel/stove type, kitchen area ventilation, quantity of fuel, age, gender and time spent near the cooking area) influencing spatial and temporal patterns within and between households/ individuals across world regions. In communities that heavily rely on solid cookfuels, household emission of pollutants can also be a significant contributor to ambient air [4] pollution. As a result, these communities often suffer from elevated indoor and outdoor air pollution.

Past burden of disease estimates for household air pollution (HAP) related to solid cookfuel combustion have relied on categorical exposure indicators such as use of solid vs. clean fuels[3,14]. Although it is known that such simple binary comparisons are imperfect as indicators of exposure differences, they had the advantage of fitting with the available epidemiological results, which used these same metrics. As few health studies in these settings had been able to simultaneously perform quantitative pollution measurements, there were no exposure-response functions available for HAP even if measured exposures had been available for a burden of disease assessment.

The field has progressed substantially, however, since the last Comparative Risk Assessment (CRA) for the Global Burden of Disease (GBD) 2000[15]. There are now a small but growing number of HAP exposure-response studies [16,17]. In addition, a set of Integrated Exposure-Response (IER) curves have been developed to link combustion particle exposures across several orders of magnitude (ranging from those due to ambient air pollution to those from active smoking, with secondhand tobacco smoke being intermediate) for specific disease end-points [18]. HAP exposures typically seem to lie between those for secondhand tobacco smoke and active smoking, with fewer exposure-response studies for disease end-points, as compared to studies for the other combustion particle sources [19]. These IERs provided the opportunity for several types of analysis for the new CRA, as part of the GBD 2010 assessment, which were not possible previously:

• HAP epidemiology studies could be used to further refine the IERs by better pinning down risks in the intermediate exposure range

• IERs could be used to determine the full risks of HAP using a low counterfactual (referred to as Theoretical Minimum-Risk Exposure Distributions in GBD-2010) level equivalent to using clean cookfuels such as gas – parallel to that used in the CRA for the CRA calls it "ambient" air pollution [4].

• IERs for diseases for which there were no available HAP studies could be used to estimate risks for HAP exposures by interpolation.

All of these activities, however, required that estimates of the actual HAP levels experienced by large populations be made, as just knowing type of fuel used, would not be sufficient.

The task of performing large numbers of household measurements around the world to accurately represent the hundreds of million households that currently use solid cookfuels would be prohibitively expensive and too time consuming to be practical. Given the heterogeneity in exposures and the resource intensiveness of such measurements, there was thus a need to develop and validate models to predict average HAP exposures in relation to household variables, information on which is often available from national surveys (or can be more easily collected using questionnaires). Exposures in urban outdoor environments have been modeled for use in disease burden assessments and policy-relevant impact studies including in developing countries [20-22] but few such modeling attempts have been made for estimating HAP exposures in relation solid cookfuel use, an exposure dominated by rural indoor environments of developing countries.

In this paper, we report results from one of first such modeling exercises that estimates average household concentrations of PM_2.5_ from solid cookfuel use by state and nationally for India, on the basis of quantitative air pollution measurements and information on household variables from multiple states. The focus of the paper is on development of models to estimate state and national average household concentrations in relation to HAP resulting from solid fuel use and not to attempt to estimate accurately, the situation in individual households. We used measurements in four states to develop and validate the model and then used national household survey data in the model to derive estimates for the rest of the country.

## Methods

We monitored 24-hr household (kitchen and living area) concentrations of PM_2.5_ in 617 rural households from 4 states in India on a cross-sectional basis. We then, developed and validated log linear regression models that predicted household concentrations as a function of multiple, independent household variables and subsequently generated state and national estimates using “household survey data” from The Indian National Family and Health Survey (2005)[23] in three stages as described below.

### Stage 1: Household monitoring for PM_2.5_

#### ***Selection of states and households for air pollution monitoring***

Six hundred and seventeen households in four geographically and culturally distinct states (Central-Madhya Pradesh (MP), South-Tamil Nadu (TN), North-Uttaranchal (UA) and East- West Bengal (WB)) of India, were recruited between November 2004 and March 2005 to perform household measurements. The choice of states was made primarily to provide a representative basis for the model. Selection of households across the country to generate a representative, measured national estimate was not feasible on account of financial and logistic constraints.

Multi-stage sampling was used to randomly select two districts from each state and three villages from each district. Approximately 25 households were selected by stratified random sampling based on fuel and kitchen type, in each village resulting in around 150–155 households from each state. Each village encompassed as many as several hundred households. To select the study households, the field team first conducted a rapid assessment of all households in the village. The team members went to each household and asked several short questions, including ones about primary fuel type and kitchen type. After the completion of the rapid assessment, a stratified random sample – based on fuel and kitchen type – of twenty five households was drawn. The following day, these households were invited to participate in the study. Urban households could not be included (we elaborate on this, in the discussion section).

Informed consent was obtained from all study households prior to any assessments. The protocols for measurements were approved by the human subjects committees of Sri Ramachandra University and The University of California, Berkeley. All household assessments including questionnaire administration and air pollution measurements were performed shortly after recruitment and simultaneously in the four states using four field teams. Field teams were trained jointly by the core investigators prior to deputing the teams for field work. A manual containing standard operating procedures was provided to all field team members for respective data collection tasks. Field data collection was completed between November 2004 and March 2005.

#### ***Measurement of PM_2.5_ concentrations in multiple household micro-environments***

24 ±2-h PM_2.5_ concentrations were measured in the kitchen and living area microenvironments using UCB Particle and Temperature (PATs) monitors, in all study households. Gravimetric instruments (portable constant-flow SKC pumps Model 224-PCAR8, SKC, Eighty-Four, PA, USA) were co-located with the UCB-PATs in a subset (10%) of the study households for validation.

Instruments were placed in the kitchen area or living area according to the following standard protocol: (1) approximately 100 cm from the stove (for kitchen area measurements) (2) at a height of 145 cm above the floor (as close as possible to the primary sleeping or sitting area for living area measurements) and (3) at least 150 cm away (horizontally) from doors and windows, where possible (for outdoor kitchen areas we used only the first two criteria). (Note: The living area was defined as the room outside of the kitchen area where household members spend the most time; it was typically a common multipurpose area and sometimes a separate bedroom. In households with a single common area used for cooking and sleeping, a separate living area could not be defined and measurements were taken only in the kitchen area as per above mentioned criteria).

UCB-PATs were used as per validated methods published previously [24,25]. Briefly, monitors were calibrated with combustion aerosols (e.g. wood and charcoal) and against temperature in the laboratory before being used in the field. Particle coefficients were derived for each instrument in the field through co-location of UCB-PATs monitors and gravimetric samplers in around 15% of households (n = 96). All UCB-PATs were zeroed in a Ziploc bag for a period of 30 to 60 minutes before and after deployment. Particle and temperature coefficients along with the results from zeroing were subsequently used in the data processing algorithm. After monitoring, all data files were batch processed using a customized software package developed for this device. This process produced a master data sheet, which was manually scanned for errors before creating an individual .csv file for each monitoring period.

Gravimetric PM_2.5_ samples were collected using methods published previously [8]. Briefly, samples were collected using a BGI triplex cyclone (scc1.062, Waltham, MA) in portable constant-flow SKC pumps (Model 224-PCAR8, SKC, Eighty-Four, PA, USA) equipped with a 37-mm diameter Teflon filter (pore size 0.45 μm also supplied by SKC) at a flow rate of 1.5 l/min. Filters were weighed using a Thermo Cahn C – 34 Microbalance (Thermo Scientific, Waltham, MA, USA) at Sri Ramachandra University and a Mettler Toledo-MT5 balance (Mettler, Greisensee, Switzerland) at The Energy Research Institute in New Delhi. Both balances operated at a resolution of 0.1 μg and were used according to the same standard operating procedure. All filters were conditioned in a temperature and relative humidity controlled room before weighing. Approximately, twenty percent of the gravimetric samples (collected from 96 households) were paired with field blanks (n = 18); none of the pre- and post- field blank weights differed by greater than 0.003 mg.

### Stage 2: Development of models to estimate household concentrations of PM_2.5_ on the basis of household determinants

Questionnaires were administered in all study households to collect information on a range of household variables. This primarily included physical variables likely to directly influence household concentrations such as fuel type, kitchen location, stove type, ventilation, fuel quantity and cooking duration. Information on indicators of other sources of indoor emission of particulate matter were also captured by recording use of solid fuels for heating, indoor smoking, number of hours without electricity (indicative of use of kerosene based lamps for lighting) and use of incense or mosquito coils. Variables likely to indirectly influence concentrations such as house type, ethnicity, income as well as behavioral variables such as meal type, type of cooking tasks etc. were collected by a larger socio-demographic survey conducted in the same villages by another team of collaborators but could not be included for analyses in this paper. We first developed models to estimate kitchen area concentrations (from measurements conducted in 617 households) in relation to these variables. Most household variables related to cookfuel use are likely to directly influence kitchen area concentrations, with living area concentrations in turn, being influenced by respective kitchen area concentrations. We therefore developed regressions equations for the relationship between kitchen and living area concentrations (from paired measurements in 427 households) in order to be able to derive the living area from measured /modeled kitchen concentrations. We describe the procedures for modeling the kitchen and living area concentrations separately in greater detail below.

#### ***Estimation of kitchen area concentrations***

We developed multiple regression models to relate the measured kitchen area concentrations of PM_2.5_ to categorical and continuous household variables. A Box-Cox procedure was used to select the optimal transformation of the dependent variable. One way Analysis of Variance (ANOVA) models were fit to each of the categorical and continuous predictors; predictors which led to a significant F-test(p < 0.05) were selected for inclusion in the multiple regression model resulting in inclusion of fuel type, kitchen type, kitchen ventilation, state (a proxy for geographical location) and cooking duration as primary model variables.

Fuel type (labeled as “Fuel” in the model) was classified as wood, dung, kerosene and LPG. (Note: fuel type refers to use of these fuels as the primary fuels during the monitoring period and may not reflect average fuel use in these households). Kitchen type/location (labeled as “Kit” in the model) was classified as outdoor kitchen (ODK), separate (often semi-enclosed) outdoor kitchen (SOK), indoor kitchen partitioned from the rest of the living area (IWPK) and indoor kitchen without partitions (IWOPK) i.e. common living and cooking areas. Kitchen a ventilation (labeled as “Vent” in the model) was classified as good, moderate and poor on the basis of self-reported availability of windows, ventilation, open eves, and the presence of chimneys and fans inside the kitchen area. The 4 states were assigned to one of four geographic regions (labeled as “Reg” in the model) viz. Uttar Pradesh (North), West Bengal (East), Madhya Pradesh (Central) and Tamil Nadu (South) respectively. Information on kerosene lamp use, mosquito coil and incense usage was collected from households but the large number of missing observations precluded their use in the model. Stove type added no additional information over fuel type as nearly all solid cookfuels were used traditional stoves (simple 3 stone fires or stoves built by the household using locally available materials including mud, plaster or metal) and was therefore excluded from analyses. Accordingly, the following regression model was fitted to the data:

(1) $$\begin{array}{ll} E\{\text{log}\left(PM_{2.5}\right)\} & =\beta_{0}+\beta_{F1}I\left(\text{Fuel}=\text{Kerosene}\right)+\beta_{F2}I(\text{Fuel}=\text{Dung})+\beta_{F3}I(\text{Fuel}=\text{Wood})\\ & +\beta_{K1}I\left(\text{Kit}=\text{SOK}\right)+\beta_{K2}I(\text{Kit}=\text{IWPK})+\beta_{K3}I(\text{Kit}=\text{IWOPK})\\ & +\beta_{V1}I\left(\text{Vent}=\text{Moderate}\right)+\beta_{V2}I(\text{Vent}=\text{Poor})+\beta_{\mathrm{CH}}(\text{Cooking hours})\\ & +\beta_{R1}I\left(\text{Reg}=\text{East}\right)+\beta_{R2}I(\text{Reg}=\text{West})+\beta_{R3}I(\text{Reg}=\text{South}) \end{array}$$

where *I(X = L) = 1*, if the categorical variable *X* assumes the level ‘*L*’, else 0

Reference categories included “LPG” for fuel, “outdoor kitchen” for kitchen type/location, “good” for ventilation and “North” for region respectively.

#### ***Estimation of living area concentrations***

Most household variables related to cookfuel use are likely to directly influence kitchen area concentrations, with living area concentrations in turn, being influenced by respective kitchen area concentrations. We therefore examined the relationship between kitchen and living area concentrations in paired measurements in order to be able to derive the living area from the kitchen concentrations.

Since the co-relation between measured living area and kitchen area concentrations was not linear, we for the paired kitchen area- living area measurements,

(2) $$\text{log}\left(\frac{L}{K}\right)=\alpha +\beta \times \text{log}\left(K\right)$$

where, L = 24-h living area PM_2.5_ concentration; K = 24 h- kitchen area PM_2.5_ concentration.

Expressing equation 2 as *L* = *δK*^1 + *β*^where *δ* = *e*^*α*^ and applying the values of δ = 0.147 and *β* = -0.680 obtained from the regression, living area room concentrations were finally estimated by equation 3 below,

(3) $$L=0.147\times K^{0.32}$$

Modeled estimates for living area room concentrations were thus derived by first, applying equation 1 to estimate kitchen area concentrations as a function of household determinants and subsequently applying equation 3 to derive living area concentrations, as a function of the respective estimated kitchen area concentrations.

Finally, correlations between measured vs. modeled values were estimated using Pearson’s correlation coefficients.

### Stage 3: Generation of state and national estimates for household concentrations

The process of generating state and national estimates using information on household variables required matching the variables from the study household questionnaires to the variables in the much larger national Indian NFHS 2005 survey (while recognizing that national surveys may not be able to capture household information at the same level of detail). Three of the five significant predictor variables for the model (primary fuel use, kitchen (type)/location and geographical region) were identical in both (i.e. study questionnaire and the Indian NFHS) datasets. Information on other two (cooking duration and kitchen ventilation) however was only available in the study dataset and was not captured in the Indian NFHS survey. We thus had to impute these values for the Indian NFHS dataset as follows.

We imputed cooking duration by linear regression of cooking hours with number of household members and type of fuel in study household dataset as

(4) $$E\;\left\{\text{Cooking hours}\right\}=\alpha +\beta \;\left(\text{No.of family members}\right)$$

Similarly, a polytomous regression model was used to impute kitchen area ventilation in terms of living room ventilation and kitchen (type)/ location allowing for possible interactions as

(5) $$\;\begin{array}{l} E\;\left\{\text{Kitchen ventilation}\right\}\\ \;=\alpha +\beta 1\;(\text{Living room windows})\\ \;+\beta 2\;(\text{Kitchen type})+\gamma (\text{Living room windows})\\ \;\times \;\left(\text{Kitchen type}\right) \end{array}$$

Once information on all significant predictor variables (actual or imputed) was assembled for the Indian NFHS 2005 household data set, coefficients from the multiple regression equation (1) were then applied to estimate household concentrations. Finally, predicted household concentrations were combined to generate state and national estimates using the state and national sampling weights used by the Indian NFHS.

### Stage 4: Assessing model accuracy through k-fold cross validation and bootstrapping

We applied cross validation and bootstrapping methods to estimate the accuracy of models developed in earlier stages. We first performed a k-fold cross validation for the household model (described in Stage 2) by excluding households from each of the 24 villages (~25 households) sequentially. The 24-fold cross-validation (using the log transformed 24 hr kitchen concentration dataset) provided an overall correlation coefficient between modeled and measured values.

Bootstrapping was then used to estimate the standard error of prediction for the national model (described in Stage 3). To compute the bootstrapping standard error of the kitchen area PM_2.5_ estimates, we first generated 200 constructed datasets (replicates) of PM_2.5_ as $\text{log}\left(P{\hat{M}}_{2.5}\right)\sim \text{Normal}\;(µ=X\hat{\beta },\sigma^{2}={\hat{\sigma }}_{e}^{2}$; where X refers to the vector of all the predictors in a household. Each constructed dataset was required to be of the same size as the original data based on estimated parameters and empirical predictors. The model was applied on each of the 200 constructed datasets (the estimates started to converge after application on 100 replicates and was doubled to allow an additional margin for stability) to obtain the empirical standard deviations of each parameter along with error variance. We used the empirical standard deviation of error variance, considered to be the standard error to obtain the bootstrapping standard error of predicted PM_2.5_ concentrations.

## Results

### PM measurements

**Figure 1:**
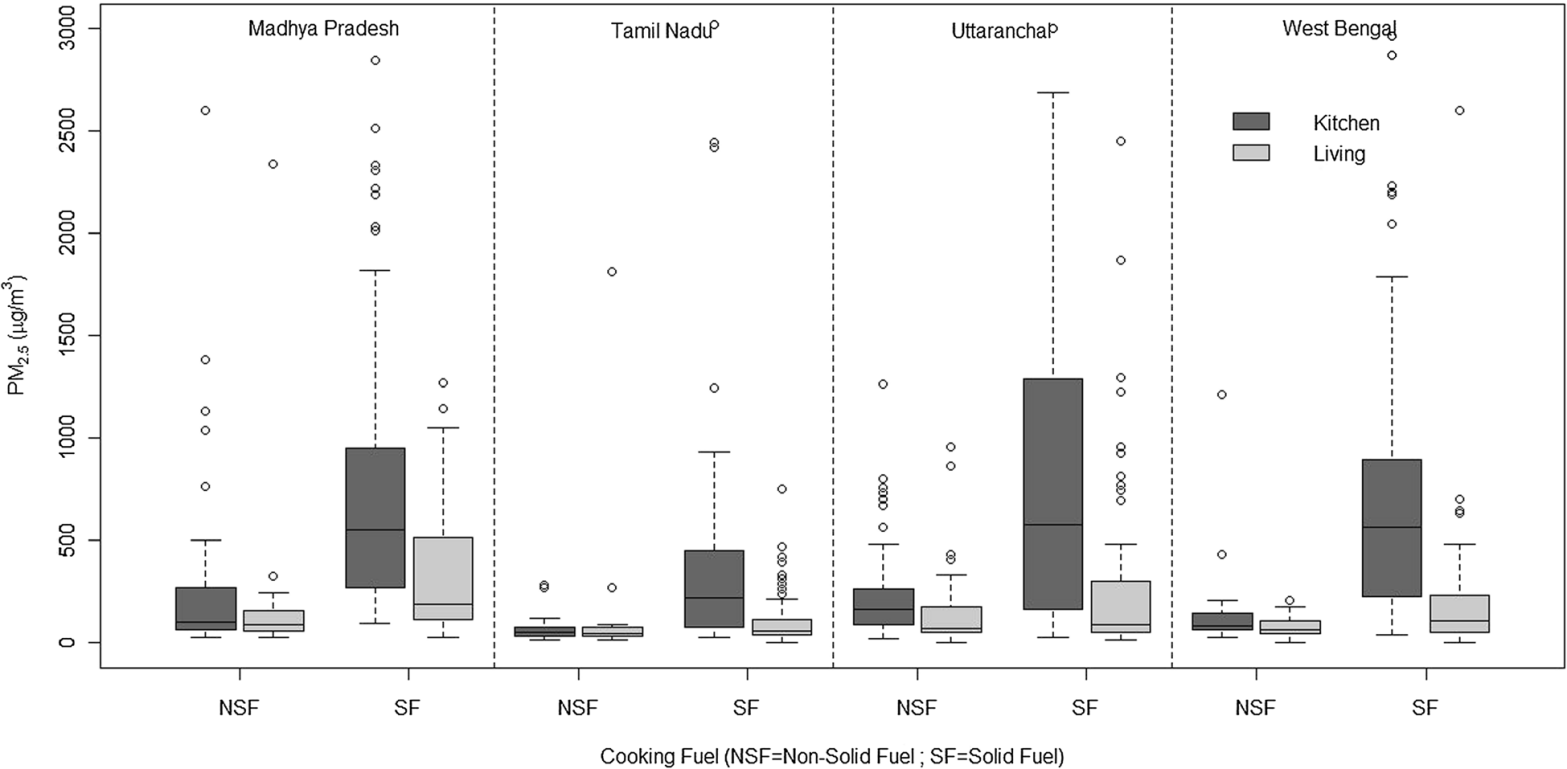
**Box plots showing the distribution of 24 hr PM **_**2.5 **_**concentrations in the kitchen area and living area areas in study households across 4 states (Note: NSF indicates use of kerosene and/or LPG as the primary fuel).**

Of the 617 households recruited, measurements covering the full 22–26 h period were obtained in 528 households. Descriptive results and the distribution of 24-h PM2.5 concentrations across the 4 states are shown in Table 1 and Figure 1 respectively. Wood was the most common solid cookfuel used. Dung use was rather uncommon except in West Bengal. Nearly all solid cookfuel users, used traditional (3-stone, mud or clay) stoves with occasional improvisations such as a raised mantle or chimney. Although higher backgrounds in the community may account for high concentrations recorded in LPG users, the large difference between kitchen and living area PM_2.5_ concentrations in these households, suggest that there may have been some residual use of other solid or kerosene fuels. This may however be the case for a minority of such households (as suggested by the larger differences in the mean as compared to the median values). We did not record any use of “cleaner” (often termed “improved”) combustion cook stoves using biomass or coal in the study areas and during the monitoring period (these were uncommon in Indian households during this period).

**Table 1:** **24hr- PM**_**2.5 **_**(μg/m**^**3**^**) concentrations (5**^**th **^**to 95**^**th **^**percentile) in relation to household variables in the 4 states**

**Predictors**	**Level**	**PM**_**2.5**_**(μg/m**^**3**^**) in kitchen area**			**PM**_**2.5**_**(μg/m**^**3**^**) living area room**		
		**(N = 474 after exclusion of 54 households as outliers)**			**(N = 427 after exclusion of 44 households as outliers)**		
		^**†**^**N**	**Mean (SD)**	**Median**	^**†**^**N**	**Mean (SD)**	**Median**
*Cooking fuel	LPG	103	179 (219)	100	91	95 (77)	72
	Kerosene	41	254 (317)	100	19	98 (95)	61
	Dung	59	741 (539)	621	55	190 (176)	115
	Wood	262	590 (575)	386	209	157 (167)	87
*Kitchen area location	ODK	56	560 (468)	473	57	167 (169)	91
	SOK	213	508 (552)	277	210	142 (146)	87
	IWPK	107	371 (509)	177	94	132 (143)	79
	IWOPK	92	536 (557)	330	16	144 (185)	81
*Ventilation	Poor	129	638 (647)	376	84	186 (173)	113
	Moderate	196	454 (513)	259	157	130 (150)	73
	Good	144	398 (421)	236	137	120 (112)	84
*Region	North	122	512 (549)	252	93	138 (155)	72
	East	130	531 (529)	345	118	144 (142)	94
	West/Central	138	549 (568)	350	86	200 (171)	146
	South	84	283 (413)	128	86	97 (114)	51
Heating	No	349	517 (558)	278	288	151 (154)	92
	Indoor	91	443 (496)	220	67	130 (150)	67
	Outdoor	26	256 (304)	119	23	90 (81)	50
Indoor smoking	No	7	500 (643)	102	20	94 (94)	48
	Yes	93	347 (633)	103	50	102 (133)	50
*Family size	≤ 4	138	457 (570)	242	166	141 (156)	75
	>4	295	509 (555)	277	203	146 (146)	91
*Cooking hours	≤ 4 hrs	278	392 (443)	218	228	121 (124)	75
	> 4 hrs	185	641 (627)	398	143	177 (176)	115
*Numbers of hrs without electricity	≤ 2.5 hrs	107	488 (584)	245	85	153 (163)	93
	>2.5 hrs	111	681(620)	510	82	206 (189)	136
Other PM sources	No	56	536 (492)	332	39	189 (189)	128
	Yes	162	514 (563)	275	110	160 (148)	117

†: No of Households;*: Statistically significant in one way ANOVA and therefore included in model; Number of hrs without electricity is used as a proxy for lighting using kerosene; other PM sources include use of incense, mosquito coils and smoking inside the house . Description of kitchen area types may be found in the main text accompanying equation 1. [Number of households recruited = 617; Valid kitchen measurements in N = 528; Valid living room measurements in N = 427; Number of kitchen measurements after exclusion of outliers = 474; Number of living area measurements after exclusion of = 427; Data shown does not include households on which the respective variables were unspecified (cooking fuel = 9, kitchen location =6 , ventilation =5, heating = 8); unknown(indoor smoking = 374) and/or; unavailable(family size = 41, cooking hours = 11, number of hrs without electricity = 256 or presence of other PM sources = 256)].

The measured mean 24-hr concentration of PM_2.5_ in solid cookfuel using households ranged from 163 μg/m^3^ (95% CI: 143,183; Median 106; IQR: 191) in the living area to 609 μg/m^3^ (95% CI: 547,671; Median: 472; IQR: 734) in the kitchen area. The difference between 24-h kitchen area concentration and corresponding living area room concentration was statistically significant in solid cookfuel using households but not in LPG and kerosene using households. Similarly, while both kitchen area and living area concentrations varied with household kitchen configuration amongst solid cookfuel users, such differences were insignificant amongst LPG and kerosene users. This is not surprising considering LPG and kerosene was almost always used in indoor kitchen areas while solid cookfuels were used across multiple configurations of indoor and outdoor kitchen areas. (This observation had important implications in the model, as explained later). Measured 24-h kitchen area and living area concentrations of PM_2.5_ across various categories of fuel and kitchen area types (Table 1) are comparable to what has been widely reported in literature in India and elsewhere in developing countries [5].

### Modeling of household concentrations in relation to household variables

**Figure 2:**
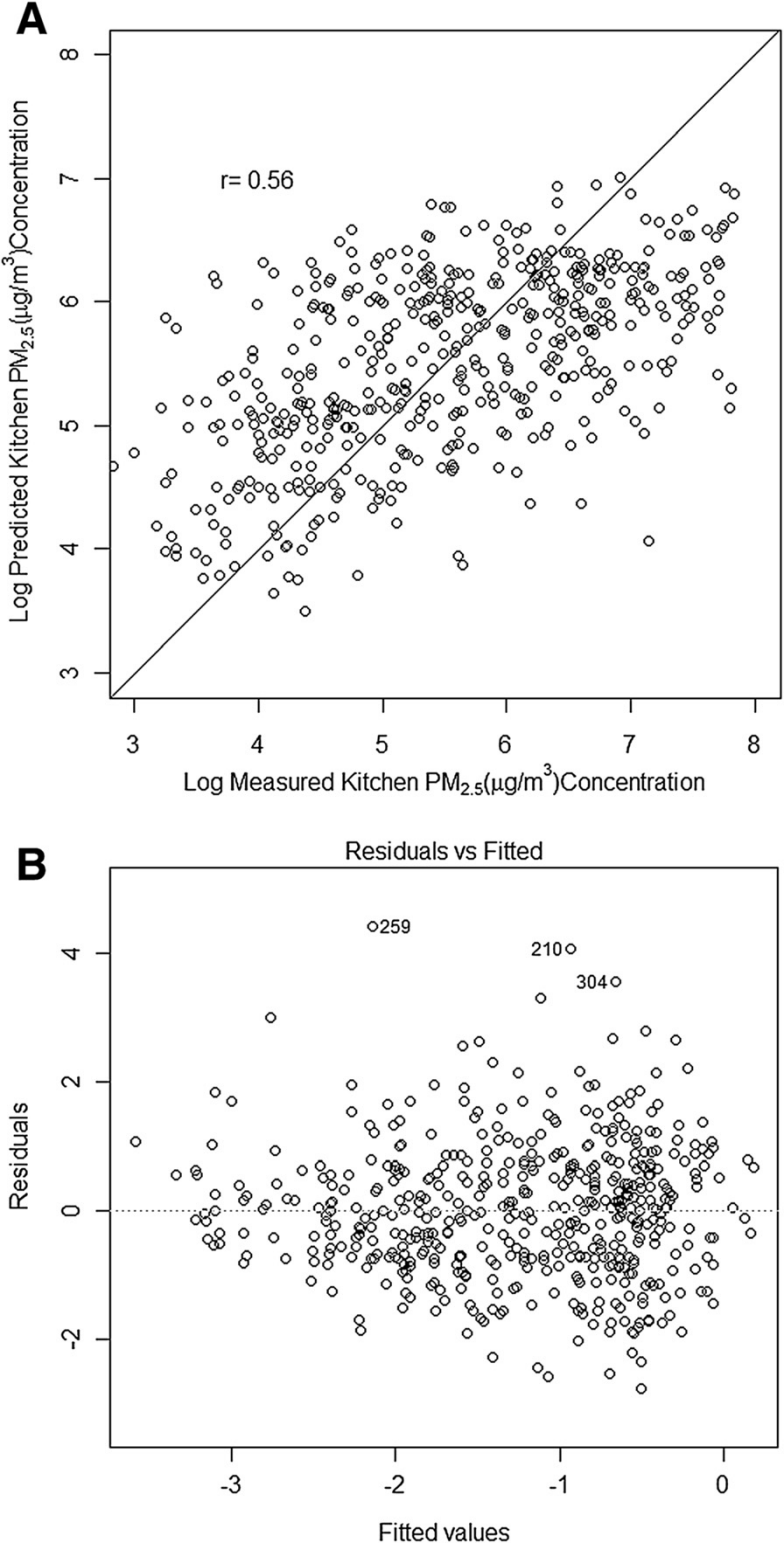
**Results from validation studies: Scatter plot of modeled vs. measured kitchen area PM **_**2.5 **_**(top) concentrations obtained from the k-fold cross validation analyses; Residual vs. fitted values (bottom) from the model.**

**Figure 3:**
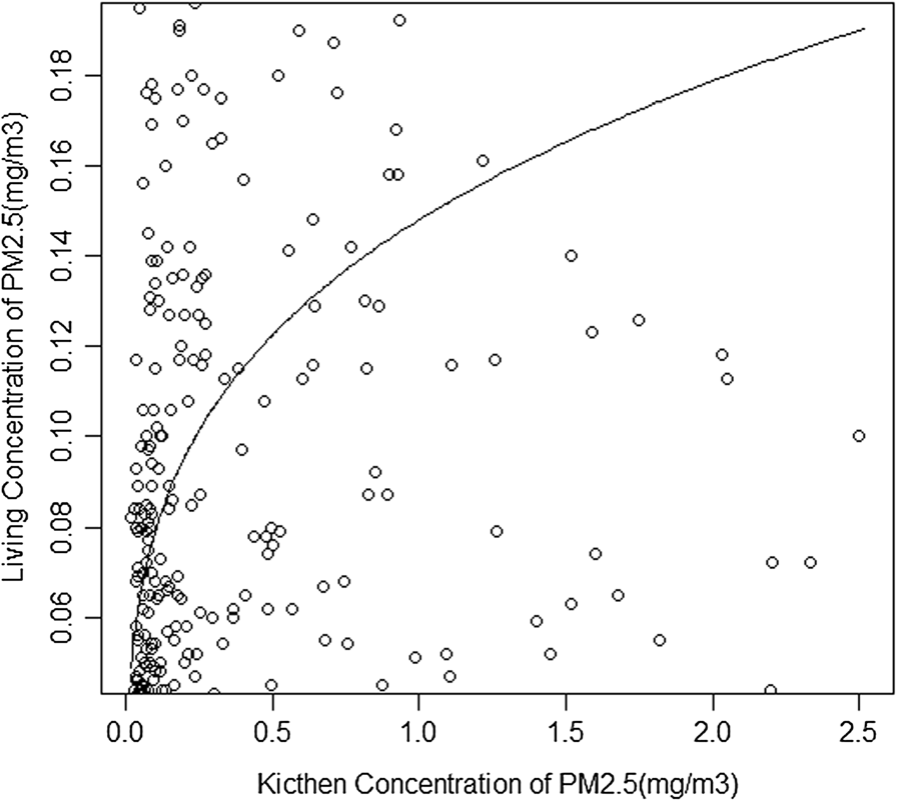
**Scatter plot of measured kitchen vs. measured living area 24-hr PM **_**2.5 **_**concentrations.**

As described in the methods, we first developed a model to estimate kitchen PM_2.5_ concentrations as a function of household variables. Since the distribution of kitchen PM_2.5_ concentrations was skewed (Figure 1) we used a Box-Cox procedure to transform the dependent variable, which led to the selection of a log-linear regression model using only values between the 5^th^ and 95^th^ percentile. The log linear regression model (equation 1), which included cooking fuel, kitchen area location, kitchen area ventilation, region (a proxy for geographical location) and cooking duration as significant predictors of 24-h kitchen area concentration of PM_2.5,_ produced an adjusted r^2^ of 0.33 (Table 2) with a fair degree of correlation (r = 0.56) between modeled and measured values upon applying cross validation methods (Figure 2). The regression model for estimating the living area concentration from the ratio of measured kitchen and living area concentrations (equation 3) produced an adjusted r^2^ of 0.72 (Figure 3). Modeled living area concentrations obtained by applying equation 3 on the respective modeled kitchen concentration (obtained from equation 1) were also fairly well correlated (r = 0.61) with measured values.

**Table 2:** **Coefficients for predictor variables from the log linear regression model (Equation** 1) **relating 24 hr kitchen area PM **_**2.5 **_**concentrations with household variables**

**Parameters**	**Estimate**	**Std. Error**	**P-value**
Intercept	-1.653	0.25008	0.000
Fuel: kerosene vs. LPG	0.194	0.17529	0.269
Fuel: dung vs. LPG	1.260	0.17166	0.000
Fuel: wood vs. LPG	0.969	0.11319	0.000
Kitchen: SOK vs. ODK	-0.389	0.1579	0.014
Kitchen: IWPK vs. ODK	-0.594	0.17807	0.001
Kitchen: IWOPK vs. ODK	-0.262	0.18316	0.153
Ventilation: moderate vs. good	-0.082	0.11155	0.461
Ventilation: poor vs. good	-0.391	0.12616	0.002
Region: east vs. north	-0.106	0.14243	0.457
Region: west vs. north	-0.071	0.12362	0.565
Region: south vs. north	-0.679	0.14001	0.000
Cooking hrs.	0.084	0.02181	0.000

Note: Predictor variables were used in Equation 1 as follows

$$\begin{array}{ll} E\{\text{log}\left(PM_{2.5}\right)\} & =\beta_{0}+\beta_{F1}I\left(\text{Fuel}=\text{Kerosene}\right)+\beta_{F2}I(\text{Fuel}=\text{Dung})+\beta_{F3}I(\text{Fuel}=\text{Wood})\\ & +\beta_{K1}I\left(\text{Kit}=\text{SOK}\right)+\beta_{K2}I(\text{Kit}=\text{IWPK})+\beta_{K3}I(\text{Kit}=\text{IWOPK})\\ & +\beta_{V1}I\left(\text{Vent}=\text{Moderate}\right)+\beta_{V2}I(\text{Vent}=\text{Poor})+\beta_{\mathrm{CH}}(\text{Cooking hours})\\ & +\beta_{R1}I\left(\text{Reg}=\text{East}\right)+\beta_{R2}I(\text{Reg}=\text{West})+\beta_{R3}I(\text{Reg}=\text{South}) \end{array}$$ (1)

*SOK* Separate outdoor kitchen, *IWPK* Indoor kitchen with partitions, *IWOPK* Indoor kitchens without partitions, *Vent* ventilation, *Reg* region. Reference categories included LPG for fuel, outdoor cooking for kitchen, good for ventilation and South for region.

### Generation of state and national estimates for household concentrations

**Figure 4:**
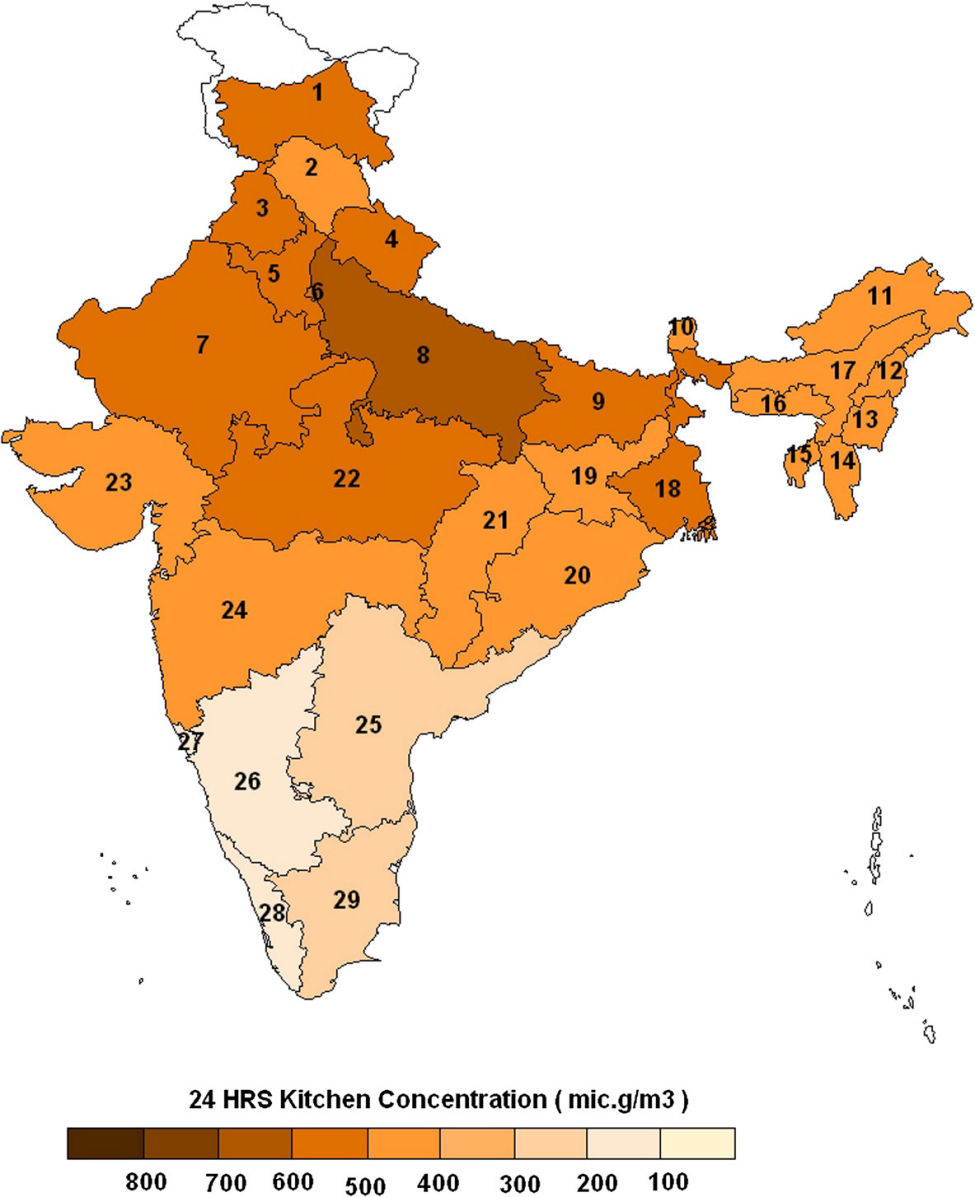
**Weighted state estimates for average 24 hr kitchen area concentrations of PM **_**2.5 **_**for all solid- fuel-using households in India (Note: Solid-fuel-using households include both urban and rural households.** State estimates are weighted by the percentages of rural, urban households using solid cookfuels as the primary fuel, respectively. Numbers indicate names of states as provided in Table 3).

Extrapolation of the household model to all solid cookfuel using households in India, covered by Indian NFHS 2005, resulted in a modeled estimate of 450 μg/m^3^ (95% CI: 318,640) in the kitchen area and 113 μg/m^3^ (95% CI: 102,127) in the living area, for mean 24-h PM_2.5_ concentrations. Although, we did not have urban solid cookfuel using households, in our empirical dataset, we assumed the distribution of concentrations in solid cookfuel using homes to be similar between rural and urban homes. Accordingly, the kitchen area concentrations in rural and urban solid cookfuel using households were estimated to be 455 μg/m^3^ [95% CI: 321, 646] and 430 μg/m^3^ [95% CI: 303,613] respectively. Further, the living area concentrations in rural and urban solid cookfuel using households were estimated to be 114 μg/m^3^ (95% CI: 102, 128) and 112 μg/m^3^ (95% CI: 100, 126) respectively. The overall median 24–h kitchen area concentration of PM _2.5_ in rural households using other fuels (including LPG and/or kerosene) was estimated to be 110 μg/m^3^ [95% CI: 78, 155] respectively. We however, did not estimate a household concentration for urban households using other fuels (LPG and/or kerosene). These are likely to be differentially influenced by traffic emissions and contributions from other solid cookfuel users in the community and our empirical dataset could not adequately represent these contributions. The state and national estimates of 24–h kitchen area concentration of PM_2.5_ in solid cookfuel using households are provided in Figure 4 and Table 3.

**Table 3:** **State and national estimates for 24 hr kitchen area concentrations of PM **_**2.5**_** (μg/m**^**3**^**) for solid cookfuel using households in India**

**State**^**1**^	**Population**	**%SF Use**	**24 hr PM **_**2.5 **_**kitchen area concentrations (μg/m**^**3**^**) in solid cookfuel using households ( 95% CI)**			**Weight in overall estimate for all solid- fuel-users**	
			**Rural solid-fuel- users**	**Urban solid- fuel- users**	**All solid-fuel-users**	**Rural**	**Urban**
ANDHRA PRADESH (25)	7621007	41.93	214 (154–296)	187 (135–259)	207 (150–287)	0.76	0.24
ARUNACHAL PRADESH (11)	1097968	65.79	472 (331–673)	409 (286–585)	463 (325–660)	0.85	0.15
ASSAM (17)	26655528	67.45	454 (328–629)	415 (298–578)	448 (323–622)	0.85	0.15
BIHAR (9)	82998509	79.04	514 (350–754)	505 (344–742)	512 (349–751)	0.75	0.25
CHHATTISGARH (21)	20833803	81.35	478 (345–663)	469 (339–649)	476 (344–660)	0.81	0.19
DELHI (6)	13850507	13.38	587 (396–875)	411 (292–579)	442 (310–631)	0.18	0.82
GOA (27)	50671017	35.99	191 (140–262)	119 (86–163)	173 (126–238)	0.75	0.25
GUJARAT (23)	1347668	53.66	491 (361–667)	423 (311–576)	480 (354–653)	0.85	0.15
HARYANA (5)	6077900	71.8	557 (383–814)	513 (353–749)	553 (380–807)	0.90	0.10
HIMACHAL PRADESH (2)	21144564	53.73	482 (356–653)	413 (305–559)	480 (355–650)	0.97	0.03
JAMMUAND KASHMIR (1)	26945829	57.01	508 (367–706)	427 (308–593)	501 (361–696)	0.91	0.09
JHARKHAND (19)	10143700	85.05	495 (342–716)	503 (347–730)	497 (344–720)	0.74	0.26
KARNATAKA (26)	52850562	65.75	199 (145–274)	181 (132–250)	196 (143–270)	0.84	0.16
KERALA (28)	31841374	71.71	183 (135–249)	158 (117–216)	176 (130–240)	0.73	0.27
MADHYA PRADESH (22)	2318822	57.16	512 (370–711)	502 (362–698)	510 (368–709)	0.82	0.18
MAHARASHTRA (24)	96878627	34.18	461 (340–627)	385 (283–524)	438 (323–596)	0.70	0.30
MANIPUR (13)	2293896	60.14	447 (319–628)	376 (268–528)	426 (304–599)	0.71	0.29
MEGHALAYA (16)	60348023	63.21	444 (320–618)	384 (274–541)	431 (310–600)	0.77	0.23
MIZORAM (14)	888573	35.36	463 (318–673)	331 (228–482)	446 (307–649)	0.88	0.12
NAGALAND (12)	1990036	64.66	430 (308–601)	399 (286–558)	421 (302–589)	0.72	0.28
ORISSA (20)	36804660	83.1	467 (325–671)	453 (315–653)	464 (323–668)	0.81	0.19
PUNJAB (3)	24358999	56.3	582 (390–870)	529 (355–791)	575 (386–861)	0.88	0.12
RAJASTHAN (7)	56507188	73.55	532 (384–740)	514 (368–717)	530 (381–737)	0.86	0.14
SIKKIM (10)	540851	41.58	469 (345–641)	374 (272–515)	468 (344–639)	0.99	0.01
TAMIL NADU (29)	62405679	50.85	210 (152–290)	182 (132–251)	205 (148–282)	0.80	0.20
TRIPURA (15)	3199203	77.12	472 (348–643)	429 (315–585)	467 (344–635)	0.87	0.13
UTTAR PRADESH (8)	8489349	59.87	601 (411–882)	578 (397–846)	596 (408–874)	0.79	0.21
UTTARANCHAL (4)	166197921	70.98	512 (370–711)	422 (303–589)	503 (363–699)	0.90	0.10
WEST BENGAL (18)	80176197	58.32	505 (360–710)	490 (349–690)	501 (357–705)	0.74	0.26
**India**	**1026066960**	**58.66**	**455 (321–646)**	**430 (303–613)**	**450 (318–640)**	**0.80**	**0.20**

^1^Numbers in parenthesis correspond to state locations shown in Figure 4 and are matched to state codes assigned in the Indian National Census.

95% CIs were generated using the SE estimates provided by bootstrapping.

## Discussion

While air pollution from solid cookfuel combustion produces a complex mixture of multiple solid phase and gaseous pollutants, PM remains the most frequently used indicator in health studies. Household PM measurements in rural solid cookfuel using settings of developing countries also remain difficult to perform on a large-scale. This study has generated a model to provide quantitative estimates PM levels that could be expected to be experienced by households on the average at a national and sub-national (state) scale and affords a major improvement over crude indicators such as “percent solid cookfuel use”, for burden of disease assessments. The model reported here represents a first such effort at a national scale and clearly would need to be refined as larger high quality datasets become available. We describe several strengths of the study design that enabled the model generation as well as weaknesses that limit its accuracy and/or precision.

### Strengths

a. Consistency and representativeness of measurements: Several studies, including some large-scale assessments of household air pollution, have previously been reported from India [26]. These have however been limited to multiple villages or districts within individual states with each study using a slightly different protocol for measurements and collecting household information. To our knowledge, this is the first time a multi-state study has been executed to capture regional differences. Further, since the air pollution measurements were made using standardized protocols by the same team of investigators using a common management framework, it was possible to exercise a high level of quality control and maintain homogeneity in data collection methods. Also, wherever feasible, the questions used for gathering primary data on household variables in the 4 states were matched with those available in the NFHS survey, to allow easier application in models that use information across household and national surveys. A comparison of measured household PM_2.5_ concentrations reported across other studies is furnished in Table 4.

b. Estimation of household concentrations in relation to type of cookfuel: Use of solid cookfuels makes the single largest contribution to household concentrations of PM_2.5._ Bulk of the contributions to the model fit was made by the type of fuel used, with PM_2.5_ concentrations in solid cookfuel using households estimated to be 2–4 fold higher than LPG and/or kerosene using households households (Tables 1 and 2). This has been borne out in many previous studies that show virtually all configurations of household solid cookfuel use in these settings to result in very high household concentrations. More importantly, the model provides a measure of likely concentrations experienced by other fuel (including LPG and/or kerosene) using homes in rural settings. Such (i.e. non-solid cookfuel using) households have served to provide the counter-factual levels of exposure in burden of disease estimations [15,27] in the past. The concentrations experienced in non-solid cookfuel households, however are far from being “clean” as often implied in the choice of a counter-factual exposure. Also, the model allows an application to urban solid cookfuel using households, although, this remains to be validated through additional empirical measurements.

c. Contributions from high levels of background : Rural LPG using households for e.g. may benefit from low indoor emissions but are still at risk from infiltration of outdoor air pollution originating from solid cookfuel use in the community/village. The lowest 24-h concentrations predicted by the model in southern states of Kerala and Tamil Nadu for non-solid cookfuel using households (ranging from 52-64 μg/m^3^ ) is still nearly twice as high as the WHO Interim Air Quality annual Target Value (IT-G1) for PM_2.5_ of 35 μg/m^3^[6]. The model predications are thus in agreement with measurement studies that record high concentrations in (so-called) cleanfuel-using households in settings with a high prevalence of solid cookfuel use[28,29]. It also points to the imminent need to address the contributions of the community outdoor concentrations to household exposures, and to (possibly) take into account multiple fuel use.

d. Contributions from other household determinants: Since the model can address the contributions of multiple independent predictors simultaneously, this affords a major improvement over individual studies that make measurements in relation to only one or few variables. For example, the model predicts a higher concentration for outdoor kitchen areas as compared to indoor kitchen areas (Table 2) for rural households. This may seem counterintuitive. However, this is to be expected if one accounts for the exclusive use of outdoor kitchen areas by biomass users while all LPG use occurs in indoor kitchen areas. Use of biomass in outdoor kitchen areas as opposed to indoor kitchen areas results in lower concentrations, but at the same time the contributions from kitchen area configurations are negligible for LPG users, as has been verified by measurements in this and previous studies[8,30-32]. The study has also generated a separate model to estimate living area concentrations from kitchen area concentrations in solid cookfuel using households, examining the ratio of living area to kitchen area concentrations in relation to kitchen area concentrations. While, dispersion from the kitchen area (the source) could be expected to influence living area concentrations, to our knowledge, no studies have attempted to model the same. Having an estimate of kitchen area and living area concentrations greatly improves the ability to perform exposure reconstructions in conjunction with time-activity budgets of populations (as is being performed with this dataset).

e. Generation of a population exposure estimate for use in Integrated Exposure Response (IER) curves in GBD-2010 assessment: As mentioned in the introduction, recent refinements in burden of disease assessment methodologies for GBD-2010 require a quantitative estimate of population exposure to be able to use IERs for relative risk estimation of various disease endpoints associated with ambient and household air pollution. The generation of a national estimate for India fulfilled this important requirement, while providing an approach for application in other countries. India had some of the largest measurement datasets available together with national survey information. GBD 2010 therefore used the household concentration estimates reported in this paper together with estimated ratios between daily average personal exposures and kitchen concentration from available published studies to arrive at personal exposure estimates for population subgroups including women, men and young children. Exposure estimates obtained thus, were used in IERs developed for estimation of relative risks for acute lower respiratory infections in children, interstitial heart disease (IHD) and stroke in GBD 2010 [4]. With very few studies currently informing the association between HAP and cardiovascular disease (CVD) endpoints, generation of the average HAP exposure estimate was especially critical in estimation of the attributable burdens for CVD through use of the IERs in the GBD-2010 assessment.

f. Application in future health studies: The model provides national and state estimates and could potentially be used to also provide aggregate estimates at the district or village levels using other sources of survey data including the Indian Census. This has important implications for use of secondary health data in future epidemiological investigations which are also often aggregated at the village/district/state level.

**Table 4:** **Comparison of reported 24 hour household area concentrations of PM **_**2.5 **_**across studies from various WHO regions**

**WHO region**	**Country**	**Primary author**	**Year**	**N**	**Reported 24 hr kitchen area concentrations (μg/m**^**3**^**)**		**N**	**Reported 24 hr living area concentrations (μg/m**^**3**^**)**
					**AM (SD)**	**GM (95% CI)**		**AM (SD)**
AFRICA	Ghana	Zhou	2011	42		60 (53–68)		
AFRICA	The Gambia	Dionisio	2008	13	361 (312)			
AFRICA	Pennise	Ghana	2009	36	650 (490)			
AFRICA	Pennise	Ethiopia	2009	33	1250 (1280)			
AMERICAS	Costa Rica	Park	2003	14	37 (33)			
AMERICAS	Costa Rica	Park	2003	7	58 (22)			
AMERICAS	Guatemala	Naeher	2000	9	527 (248)			
AMERICAS	Guatemala	Naeher	2001	17	868 (520)			
AMERICAS	Guatemala	McCracken	1999	15	1102			
AMERICAS	Mexico	Zuk	2006	36	693 (339)	616		
AMERICAS	Nicaragua	Clark	2011	115	1354 (1275)	913		
AMERICAS	Guatemala	Northcross	2010	138	900 (700)			
AMERICAS	Mexico	Masera	2007	33	1020	910		
EMR	Pakistan	Colbeck	2010	14	1169 (1489)		7	603 (421)
SEAR	Tibet	Gao	2009	30	178 (192)		21	103 (85–121)
SEAR	India	Dutta	2007	21	950 (1210)			
SEAR	India	Chengappa	2007	30	520 (750)			
WPR	China	Baumgartner	2011			107 (74–154)		

### Limitations

a. Unavailability of longitudinal measurements: The cross-sectional study design imposed a major limitation in that it failed to capture household level variability over time, a major reason for the modest explanatory power of the model for predicting the situation in individual households. Some parts of India can experience significant seasonal variations in household concentrations. Although the measurements were performed within a single season (between December 2004 and March 2005) across all states, single season measurements may not adequately capture variations in long-term exposures. The design served the current purpose of the model development i.e. to generate aggregate estimates for the population, future refinements would be needed before such models can be applied in epidemiological studies. Longitudinal assessments and more detailed information in household surveys can both contribute towards the same.

b. Inability to perform personal exposure and ambient concentration estimates: We could not assess ambient concentrations owing constraints of obtaining power supply in the villages. We also could not perform personal exposure measurements. We were thus unable to explore the correlation between household or ambient concentrations and individual exposures. Although exposure reconstructions in progress would address some of this concern, direct measurements of personal exposures would be needed in the future to better estimate actual exposures for various sub-groups in the population. Longitudinal studies that measure multiple household area concentrations and personal exposures for various sub-groups of household members are needed to refine the extrapolation from household concentrations to individual exposures.

c. Inadequate or imprecise information on some predictor variables: Information on several predictor variables in the household model could not be readily extracted from the NFHS dataset. The study had to impute this information from available variables. Applying equations 4 and 5 to impute information on cooking hours and ventilation, resulted in a modest adjusted r^2^ of 0.20 for cooking hours and predicted 30% of the “good”, 90% of the “moderate” and 40% of the “poor” ventilation categories respectively. Information on these variables would need to be better captured in the household surveys. Inclusion of important predictor variables in population surveys in consistent ways will also enhance the ability to interface data from individual studies with national surveys.

d. Need for extension across more states: While measurements across multiple states provided representativeness for the model, to be truly nationally representative, measurements would need to include more states. This will provide further validation for a national estimate and better describe the distribution of exposures across states.

e. Need for additional PM and other air toxics measurements: The UCB-PATS monitor does not afford the same level of accuracy as would have gravimetric measurements. Although we followed a rigorous protocol to validate the UCB-PATS measurements, and the measured levels were in good agreement with reported gravimetric results from the same states [26], larger gravimetric datasets in the future would likely enhance the robustness of the estimates. Also, while PM may be a good indicator for several health effects other air toxics may be independently associated with select health effects (e.g. CO with birth weight, PAHs with cancer etc.). Relationships between pollutants would need to be examined to make judgments about the relative efficacy of using PM as an indicator.

## Conclusions

Although in need of further refinements, the model shows substantial promise to be able to generate household concentration estimates due to cooking fuel in rural households that may be aggregated to estimate population exposures at the state or national level in India. The predictive power for estimating concentrations in individual household is modest, but at the state and national level in India, it provides substantial improvement over simple binary metrics such as solid versus non-solid cookfuel use, commonly used as exposure indicators, in HAP studies. Such a population estimate was essential to allow a linkage to the IERs in conducting the more sophisticated CRA analyses for the GBD-2010 [4]. The model estimates also add considerable strength of evidence for the need to scope and implement effective public health intervention efforts at the state and national levels. With the average concentrations experienced in households being significantly higher than health-based air quality guideline values, the results from the study indicate the need for achieving substantive exposure reductions for the population.

In the 30 years since the first set of solid cookfuel related exposure studies in rural households of developing countries were reported [33], progress on developing good models that are sophisticated enough to capture the heterogeneity while relying on easy to collect indicators has been slow, with only a few recent studies making significant contributions [11,12]. We hope the results presented in the study spur additional efforts to validate as well as develop newer models to address the complexities of exposure reconstruction for household air pollution at individual, local, national and global scales. Routine integration of measurement efforts with national surveys such as NFHS, LSMS and DFHS would not only allow additional refinements in the model for estimates in the future, but also allow the use of such models in monitoring and evaluation of public health efforts directed towards intervention for HAP.

## Consent

Written informed consent was obtained from all subjects who participated in the study, for the publication of this report and any accompanying images.

## Abbreviations

AQG: Air quality guidelines; CRA: Comparative risk assessment; DFHS: District Family Health Survey; GBD: Global burden of disease; HAP: Household air pollution; IER: Integrated exposure-response curve; NFHS: National family health survey; LPG: Liquified petroleum gas; LSMS: Living Standards Measurement Study; PM: Particulate matter; WHO: World Health Organization.

## Competing interests

The authors declare that they have no competing interests.

## Authors’ contributions

KB co-ordinated the design and analyses with all co-authors and took the lead in writing the manuscript. SG and BG developed the household, state and national level models, SS co-ordinated air pollution measurements, DB provided assistance with model development, NB and KRS provided the framing for study design and shaped the analyses to fit the requirements of the GBD-2010 assessment. All authors read and approved the final manuscript.
